# Type II Pfieffer misdiagnosed as Crouzon syndrome with additional features of supernumerary teeth and localized symmetrical gigantism: a case report

**DOI:** 10.1186/s13256-022-03586-2

**Published:** 2022-11-01

**Authors:** Karim P. Manji, Mariam Mngoya Massomo, Edna Samson Akyoo, McLean Abisai Luvinga

**Affiliations:** 1 Pediatrics and Child Health, Muhimbili University of health and Allied Sciences, P.O. Box 65001, Dare-es-Salaam, Tanzania; 2 Neonatal Unit, Muhimbili National Hospital, P.O. Box 65001, Dar-es-Salaam, Tanzania

**Keywords:** Type II Pfeiffer syndrome, Early diagnosis, Ethical issues, Genetic testing

## Abstract

**Background:**

Pfieffer syndrome is among the syndromes seen in the recognized variant of the FGFR2 gene. There are several conditions related to this variant and a very closely related condition is Crouzon syndrome. This case is important to report because the neonate was a delayed referral from another region, without clear counseling and information on the gravity of situation. We describe additional features , not previously described in Pfieffer or Crouzon syndrome, supernumerary teeth and localized symmetrical gigantism of thumbs and great toes on both sides. That a genetic testing is essential to further manage and counsel to avoid lost opportunities for future births. Several cases are seen in this unit annually, and there is need for a more consolidated and comprehensive counseling and genetic testing. Once early diagnosis is done and the case is recognized to be untreatable, it was avert the need to refer.

**Case presentation:**

A 2-week-old male African neonate referred from outside the region, presented with massive proptosis soon after delivery, with signs of pan-ophthalmitis and neonatal sepsis. The infant had additional multiple malformations and features initially diagnosed as Crouzon syndrome , but later confirmed after genetic testing to be Type II Pfieffer syndrome. A through clinical evaluation and genetic testing would prevent undue referral to a tertiary center, or if needed, the baby should have been referred much earlier. The uniqueness of this case is the presence of supernumerary teeth.

**Conclusion:**

A complicated, difficult to remedy case, referred to tertiary center, investigated, and sent back home with no significant intervention. Genetic test confirmed the typical findings of Pfieffer Type II. Presented for describing additional unique features of supernumerary teeth and localized gigantism and ethical challenges in management.

## Background

Pfieffer syndrome is among the syndromes seen in the recognized variant of the FGFR2 gene. There are several conditions related to this variant and a very closely related condition is Crouzon syndrome. Several similar cases are seen in this unit annually, and there is need for a more consolidated and comprehensive counseling and genetic testing. Once early diagnosis is made and the case is recognized to be untreatable or futile, it would avert the need to refer to a tertiary center

## The case

A male African baby born at term. He is the third sibling. Uneventful pregnancy and delivered normally. Mother is in early third decade and father in the later third decade. Non-Consanguineous. Referred to this tertiary neonatal unit for further management in the second week of life.

Presented with multiple congenital malformations, the most prominent of which was externally protruding eyes, with abnormal facies. Wide forehead, clover shaped head , and abnormal ears, nasal blockage, supernumerary teeth, and locally hypertrophic toes and thumbs on both sides.

## Further physical examination

**Fig. 1:**
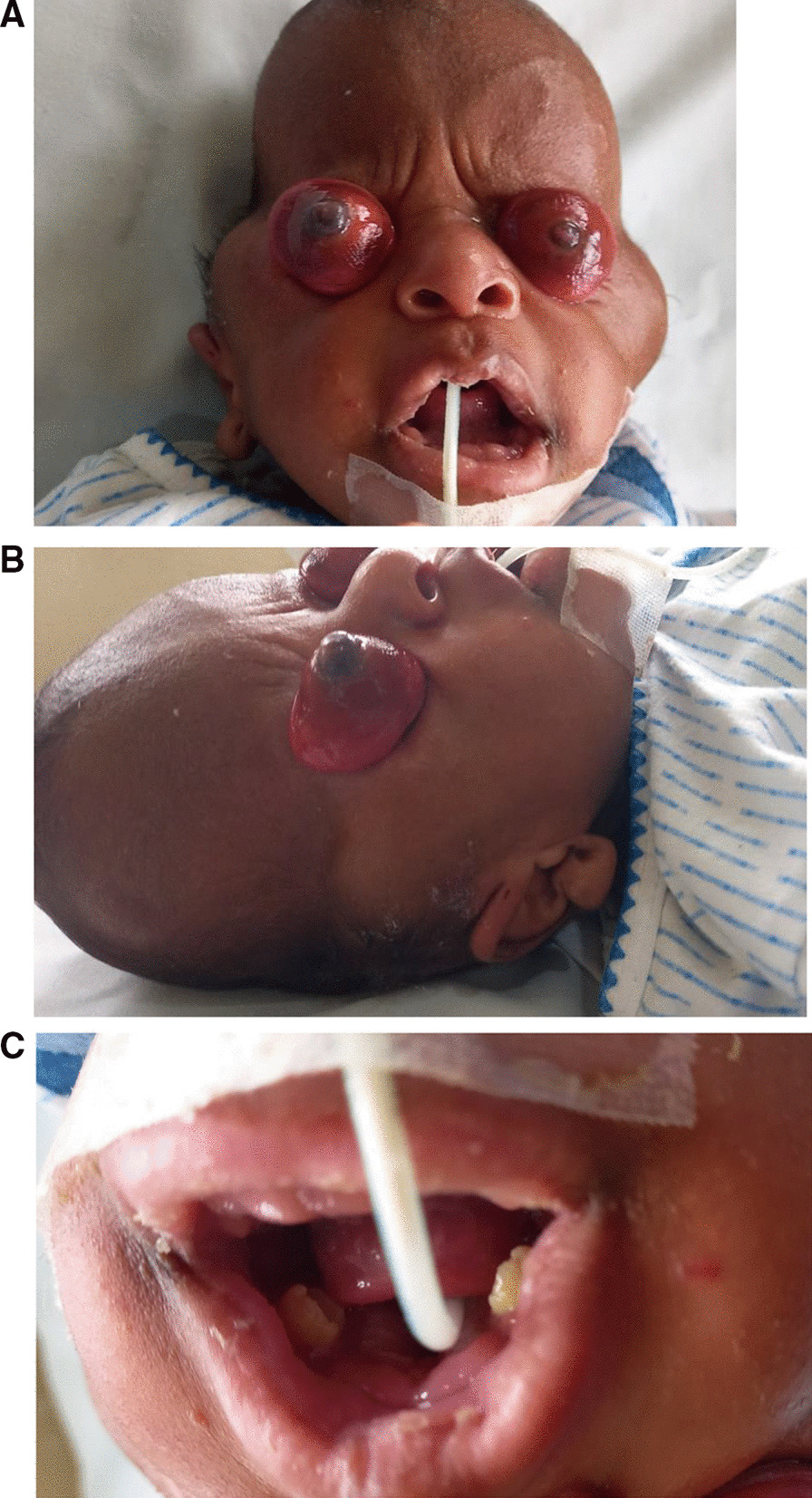
**A** Proptosis, abnormal low-set ears, clover shaped skull with proptosis and protruding temporo-zygotic areas. **B** Flattened Occiput, Pan-ophthalmitis, Low-set abnormal ears, pre-auricular sinus. **C** Supernumerary teeth

**Fig. 2:**
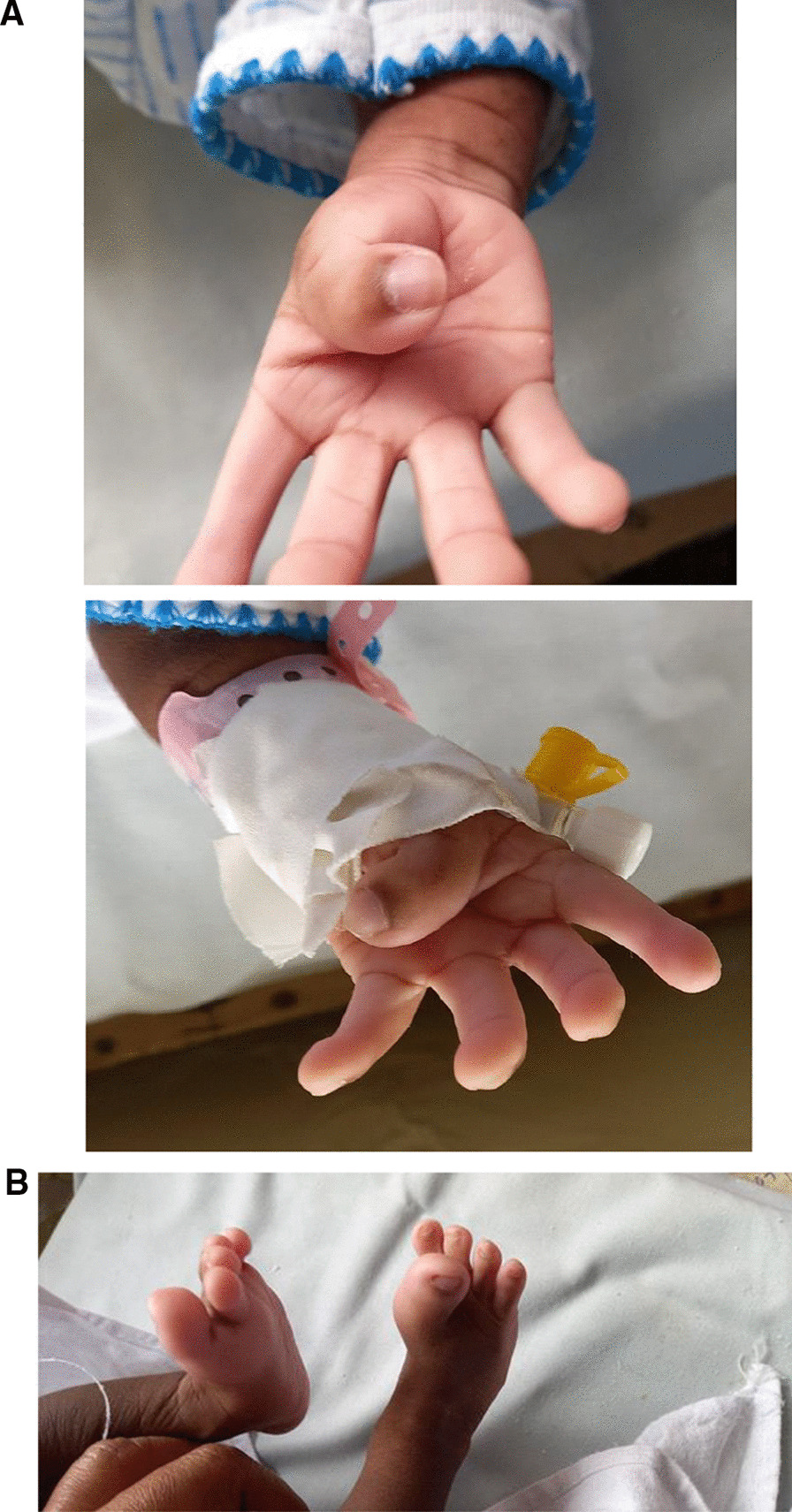
**A** Localised Gigantism of thumbs, both hands. **B** Localised Gigantism of both great toes

Proptosis, Cranio-synostosis, clover head, supernumerary teeth, localized gigantism. Severe, widely opened sutures, high arched palate, and nasal obstruction, low set ears. In short, there were abnormalities of facial appearance with proptosis, skull shape, cranial sutures with wide open from frontal to occipital, pinnae, skeletal system with kyphoscoliosis (Figs. 1A–C, 2A, B).

Cardiac examination revealed an Atrial Septal Defect (ASD) and a Ventricular Septal Defect (VSD). There were no clinical features of heart failure, and cardiologist was of the opinion of conservative management for time being.

The ophthalmologist reviewed the child, but since there was severe edema and pan-ophthalmitis, it was difficult to place sutures to close the eyelids, and due to the configuration of spine and neck, it was not possible to provide anesthesia for enucleating.

The unique features of this child were supernumerary teeth and large (localized gigantism) thumbs and great toes bilateral. These are the features not commonly described and add to medical literature particularly to differentiate between this case and the phenotypically similar Crouzon syndrome.

## Investigations

**Fig. 3:**
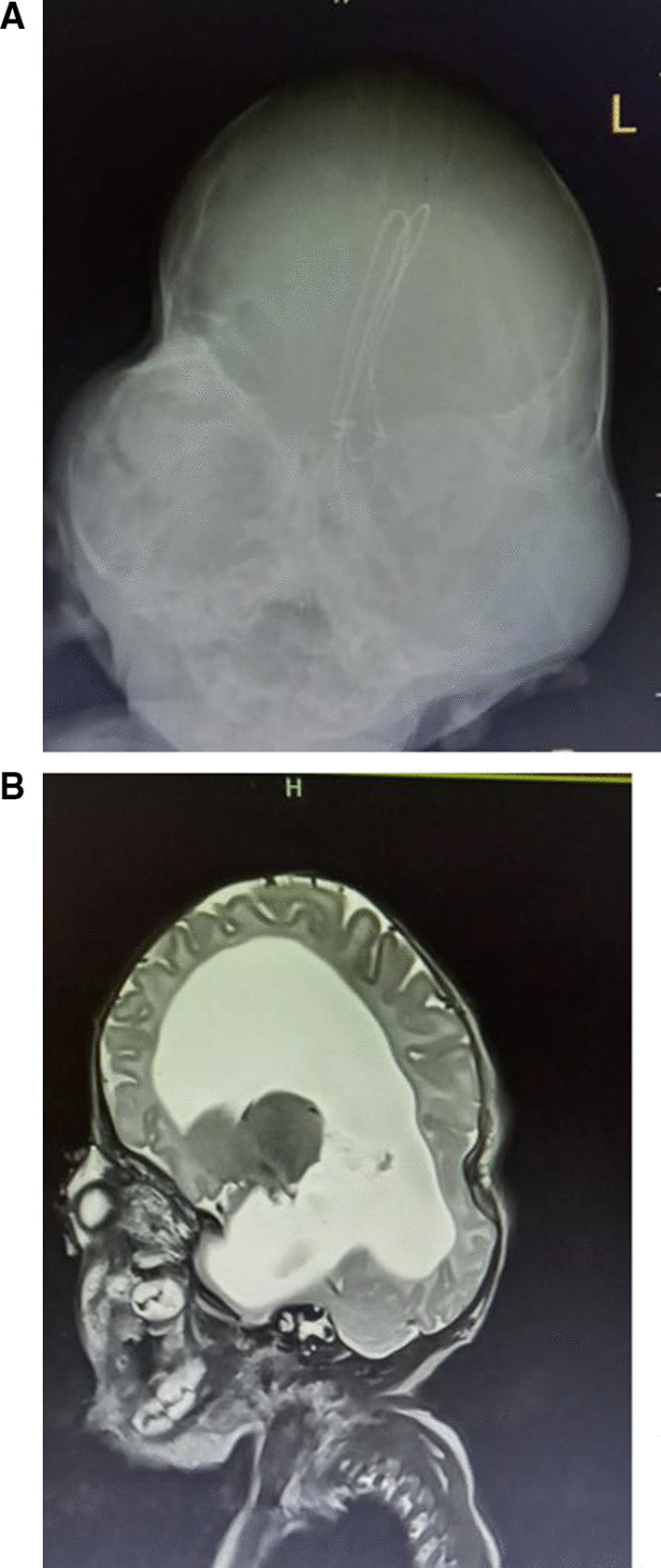
**A** Skull configuration, like a clover-leaf. **B** Ventriculomegaly, Hypoplastic Vermis, Partial agenesis of Corpus Callosum, Supernumerary teeth seen in jaw area

MRI of the Brain revealed abnormal skull configuration and prematurely fused base of skull, with ventriculomegaly and poor developed corpus callosum and vermis. The cortex was poorly configured (Fig. 3A, B)

ECHO revealed an atrial septal defect and a ventriculo-septal defect.

Genetic testing of the child was done through a genetic firm which helps to do free genetic testing (4) and a complete genome sequencing. The results showed a heterozygous pathogenic variant for Pfieffer syndrome was identified in the FGFR2 gene. The genetic diagnosis of an autosomal dominant FGFR-related craniosynostosis is confirmed. Genetic review: The dried blood spots for genetic testing was done after due consent from the mother. This was sent to Centogene in Germany, who provided free testing for our hospital patients, under our agreements. The report described the presence of FGFR2 variant c.870G>C p.(Trp290Cys). This variant causes an amino acid change from Trp to Cys at position 290. This is the typical variant for Pfieffer syndrome Type II. This variant has been described previously. Eight disorders are recognized with this FGFR mutation. These include Pfeiffer syndrome, Apert syndrome, Crouzon syndrome, Beare-Stevenson syndrome, FGFR2-related isolated coronal synostosis, Jackson-Weiss syndrome, Crouzon syndrome with acanthosis nigricans (AN), and Muenke syndrome [1–3]

### Differential diagnosis

Pfieffer Type II and Crouzon Syndrome are very closely related in their phenotype.

## Treatment

Conservative management

1. There was eye dressing and sterile patch daily.
2. Nasogastric feeds , with expressed mothers’ milk
3. Antibiotic drops for the eye
4. Lasix and Aldactone to relieve congestive cardiac failure.

Parental carrier testing is recommended to establish whether the detected variant is inherited or de novo. We recommend clinical evaluation for genotype-phenotype-correlation.

## Outcome

The child stayed for almost seven weeks at the neonatal unit. Consultations were done with ophthalmologists, neurologists and cardiologists. The neurologist was the opinion of conservative management. The child was discharged back home to the region and counseled about the gravity of the situation. Child died at home within a week of discharge. From the telephone interview, the child died of possible septicemia and multi-organ failure. She described difficult breathing, inability to feed and inability to pass urine. Due to social-cultural reasons we did not dwell further in the verbal autopsy of the child.

## Discussion

This is a genetic disorder. The anomalies could have been detected during antenatal period using an antenatal ultrasound scan for anomalies.

This units receives about 2-3 cases of similar nature annually; these are clinically very similar. It is necessary to continue to inform and educate the colleagues about the need for early referral and treatment.

Similar cases have been published by colleagues from this unit and a clinical diagnosis made, the child had bilateral choanal atresia [4].

Ten percent of neonatal teeth are supernumerary, and unusual in neonates with Crouzon, and those described in Pfieffer are usually conical and more on the incisor. These were molars and had cusps [5].

Syndactyly distinguishes Aperts syndrome from Crouzon syndrome. This baby has separate fingers, but the thumbs and toes were hypertrophied , and appeared to be like localized gigantism [6, 7]

Environmental factors include when to refer, where to refer and whether to refer at all and these overlap with the ethical dilemmas.

Ethical dilemmas in the neonatal unit include issues like these of decisions of when and where to refer. This case highlights various issues of diagnosis and management of this rare disease. Pfieffer syndrome is reported to happen in about 1: 80,000 deliveries. There was no ultrasound done [8], and on delivery immediate eye care was not provided. The baby was fed by Nasogastric tube. The eyes developed exposure keratitis and developed pan-ophthalmitis as seen in the Fig. 1A, B. Genetic counseling was not done, and the father was unable to travel to this center for further counseling. We received the results from the Centogene [9] a month later. After contacting the mother, she informed us of the child’s demise and was clearly disappointed and not willing to discuss anything further, although she did not mind her child to be reported in medical literature. for educating purpose.

Pfeiffer syndrome and Crouzon syndrome are compatible with life, and many children can live near normal life. This child has many additional malformations. A rapid intervention and multidisciplinary approach are required. The factors related to finances and travel from another region also needs to be considered.

Craniofacial dysostosis is a genetically transmitted complex disorder. The specific mutation is spontaneous and is not associated with consanguinity. The genetic testing revealed FGFR-a specific mutation in Chromosome 10. It has a variable penetrance and manifestation, and this baby has a significant gene penetrance.

### In conclusion

We provide unreported features of Supernumerary teeth and localized gigantism of toes and thumbs in Type II Pfieffer syndrome. This child presented with several ethical issues of concern, challenges in Clinical management and genetic counseling.

## Data Availability

Case report and genetic testing result attached as submission
